# Emergence and genomic characteristics of multi-drug-resistant *Salmonella* in pet turtles and children with diarrhoea

**DOI:** 10.1099/mgen.0.001164

**Published:** 2024-01-03

**Authors:** Wei Wang, Feng Liu, Hui Li, Menghan Li, Yujie Hu, Fengqin Li, Jing Xiao, Yinping Dong

**Affiliations:** ^1^​ NHC Key Laboratory of Food Safety Risk Assessment, China National Center for Food Safety Risk Assessment, Beijing, PR China; ^2^​ Division IV of Food Safety Standards, China National Center for Food Safety Risk Assessment, Beijing, PR China; ^3^​ Pharmaceutical Department, Qingdao Traditional Chinese Medicine Hospital (Qingdao Hiser Hospital), Qingdao Hiser Hospital Affiliated of Qingdao University, Qingdao, Shandong, PR China

**Keywords:** *Salmonella *Thompson, pet turtle, children with diarrhoea, multi-drug resistance, whole-genome sequencing

## Abstract

Pet turtles are a well-recognized source of human salmonellosis, posing a threat to human health, particularly children who commonly keep pet turtles. To date, the genomic characteristics of *Salmonella* among pet turtles and children has not been well described. We investigated the prevalence, antimicrobial resistance (AMR) and genomic characteristics of *Salmonella* from pet turtles in Beijing, China. In total, 9.6 % (46/480) of pet turtles were positive for *Salmonella* with *S*. Thompson being the dominant serovar (19/46) in 2019. Moreover, 80.4 % of *Salmonella* were multi-drug resistant (MDR) and 60.7 % were resistant to ampicillin, streptomycin, sulfonamides and tetracycline (ASSuT). We further compared the genomes of *S*. Thompson isolates from pet turtles (*n*=19) with those from children with diarrhoea (*n*=28) in the same region and year, most of which were sequence type (ST)26, with one novel ST7937 identified from a child-associated isolate. *S*. Thompson isolates from children with diarrhoea exhibited less resistance than isolates from pet turtles. Most MDR isolates possessed multiple AMR genes, including the AmpC β-lactamase-encoding genes *bla*_DHA-15_ and *bla*_DHA-1_ which co-occurred with the IncA/C and IncHI plasmid replicon types. To the best of our knowledge, this is the first time that the *bla*_DHA-15_ gene has been detected from *Salmonella*. Several pet turtle-associated *S*. Thompson isolates comprised phylogenetically close clusters with those from children with diarrhoea (<20 SNP differences). Bayesian analysis demonstrated that the Chinese ST26 *S*. Thompson strains had a recent evolutionary history and evolved into two major clades, with one clade acquiring various resistant plasmids. Our findings revealed the emergence of MDR *Salmonella* among pet turtles in China and provided evidence for the interspecies transmission of *S*. Thompson.

## Abbreviations

AMP, ampicillin; AMR, antimicrobial resistance; AMU, antimicrobial usage; ASSuT, ampicillin, streptomycin, sulfonamides and tetracycline; AST, antimicrobial resistance testing; BF, Bayes factors; BHI, brain heart infusion; CAZ, ceftazidime; CHL, chloramphenicol; CIP, ciprofloxacin; CTX, cefotaxime; ESBL, extended-spectrum beta-lactamase; ESS, effective sample size; gDNA, genomic DNA; GEN, gentamicin; HPD, highest posterior density; HPD, highest posterior density; IPM, imipenem; MDR, multi-drug resistant; MEM, meropenem; MIC, minimum inhibitory concentration; MRCA, most recent common ancestor; NAL, nalidixic acid; NCBI, the National Center for Biotechnology Information; PCR, polymerase chain reaction; PMQR, plasmid-mediated quinolone resistance; QRDRs, quinolone resistance-determining regions; SAM, ampicillin-sulbactam; SNP, single-nucleotide polymorphism; SPI, *Salmonella* pathogenicity island; ST, sequence type; STR, streptomycin; SXT, trimethoprim-sulfamethoxazole; TET, tetracycline; WGS, whole genome sequencing.

## Data Summary

1. All newly sequenced data in this work were deposited in the National Center for Biotechnology Information (NCBI) under the BioProject PRJNA833728.
2. All accession numbers of the publicly available genomes were available in Data Set S1, available in the online version of this article.

### Impact Statement

*Salmonella* is recognized as a natural inhabitant of the turtle’s gastrointestinal tract and contact with turtles is considered to carry a particularly high risk of infection. However, little information is available on the genomic characteristics of *Salmonella* among pet turtles in China. In this study, we analysed the antimicrobial resistance (AMR) and genomic features of *Salmonella* among pet turtles and children with diarrhoea in Beijing, China, finding significant prevalence of multi-drug-resistant (MDR) *Salmonella*, especially ESBL- and AmpC β-lactamase-producing *Salmonella,* in both hosts. SNP-based analysis revealed that *S*. Thompson from pet turtles and children with diarrhoea might arise from the same source and pet turtles may serve as important reservoirs of infections in children. Bayesian analysis of the MDR *S*. Thompson suggested that Chinese *S*. Thompson isolates have evolved divergently (such as by acquiring multiple AMR genes) to those from abroad. Owing to the potential origin in pet turtles, a One Health approach should be implemented to support surveillance whilst informing interventional strategies.

## Introduction

Non-typhoidal salmonellosis is one of the most common and widely distributed zoonotic diseases, posing a great threat to human health. It is estimated that non-typhoidal salmonellosis is responsible for approximately 93.8 million *Salmonella* infection cases and 155 000 deaths worldwide yearly [1]. The increasing antimicrobial resistance of non-typhoidal *Salmonella* to conventional antimicrobial agents (ampicillin and trimethoprim/sulfamethoxazole) and even empirical antimicrobials (amoxicillin/clavulanate, third-generation cephalosporins and fluoroquinolones) has greatly hampered the clinical treatment of human infections, particularly among immunocompromised individuals, children and the elderly [2, 3]. The resistance of *Salmonella* to third-generation cephalosporins is primarily mediated by the production of extended-spectrum β-lactamases (ESBLs) of the TEM, SHV and CTX-M types, as well as the AmpC β-lactamases of the CMY and DHA types, which are associated with different mobile genetic elements [4, 5]. These have been described not only in clinical *Salmonella* isolates but also in isolates from animals and food [5–7].

*Salmonella* can lead to human infections through direct or indirect contact with animals, which can act as reservoirs of *Salmonella* [7, 8]. As a carrier of *Salmonella*, turtles are a well-recognized source of human salmonellosis [9, 10]. Turtles are commonly considered as house pets, mostly kept by children. The latter may acquire *Salmonella* via direct or indirect contact with infected pet turtles [10, 11]. There have been several reports of outbreaks of human *Salmonella* infections involving turtles in the past decade [12–15]. However, such similar outbreaks have not yet been reported in China.

*Salmonella* Thompson (*S*. Thompson) was first reported in 1924 in Yorkshire, UK [16], and has recently become associated with human infections [17, 18]. According to the US Foodborne Diseases Active Surveillance Network, non-typhoidal salmonellosis with *Salmonella* serovars Typhimurium and Heidelberg has decreased, but the incidence of *S*. Thompson and other serovars has increased in comparison with that during 2007–2008 [19]. In Canada, a high infection rate has been observed with *S*. Thompson in the northwest region [20]. In Korea, a large-scale outbreak of gastroenteritis caused by *S*. Thompson was reported in 2018, with a total of 2207 people infected [17]. Previous reports in China have revealed that pet turtles seem to be susceptible to *S*. Thompson [9, 21]. However, there are few data available on the genetic relatedness of *S*. Thompson between pet turtles and children in China.

Over the past decade, high-resolution whole-genome sequencing (WGS) analysis has been applied to reveal the evolution and characteristics of *Salmonella* among humans and animals. Using WGS analysis, here we aimed to investigate the genomic characteristics and evolutionary relationships among isolates from pet turtles and children with diarrhoea in Beijing, China. We described the phylogeny of *Salmonella* isolates using a data set of 74 genomes collected from pet turtles and children with diarrhoea during 2019 in Beijing, China; we also investigated the distribution of antimicrobial resistence (AMR) genes, especially ESBL- and AmpC β-lactamase-encoding genes, and estimated the Bayesian divergence of *S*. Thompson genomes.

## Methods

### Bacterial isolation

A total of 480 faecal samples were collected from pet turtles in markets in Beijing, China, during 2019 on a biweekly basis using cotton swabs. All of the faecal samples were subjected to qualitative analysis for *Salmonella* using an enrichment method described in the National Food Safety Standard of China-Food Microbiological Examination, *Salmonella* (GB 4789.4–2016). In brief, faecal samples were transferred into 10 ml buffered peptone water (HopeBio), and incubated at 36±1 °C for 8–18 h. A 1 ml aliquot of the mixture was then transferred to 10 ml of tetrathionate broth (HopeBio) and selenite cystine broth (HopeBio) for selective incubation at 42±1 and 36±1 ° for 18–24 h, respectively. Loopfuls of the resulting cultures were streaked on xylose lysine desoxycholate agar (HopeBio) and Chromagar *Salmonella* (CHROMagar Microbiology), followed by incubation at 36±1 °C for 18–24 h. Suspected *Salmonella* colonies were streaked onto trypticase soy agar (HopeBio) and further incubated at 37 °C for 18 h.

Further, presumptive *Salmonella* were selected for biochemical confirmation using API 20E test identification test strips (bioMérieux), as well as for molecular identification using PCR assays targeting the *invA* gene [22]. For all of the confirmed *Salmonella* isolates, serotypes were determined with the slide agglutination test, using *Salmonella* antisera (Statens Serum Institute), according to the Kauffmann–White scheme [23]. All of the confirmed *Salmonella* isolates were stored in brain heart infusion broth with 40 % (v/v) glycerol (Land Bridge) at −80 °C. Each retained sample was represented by at least one bacterial isolate.

**Fig. 1.**
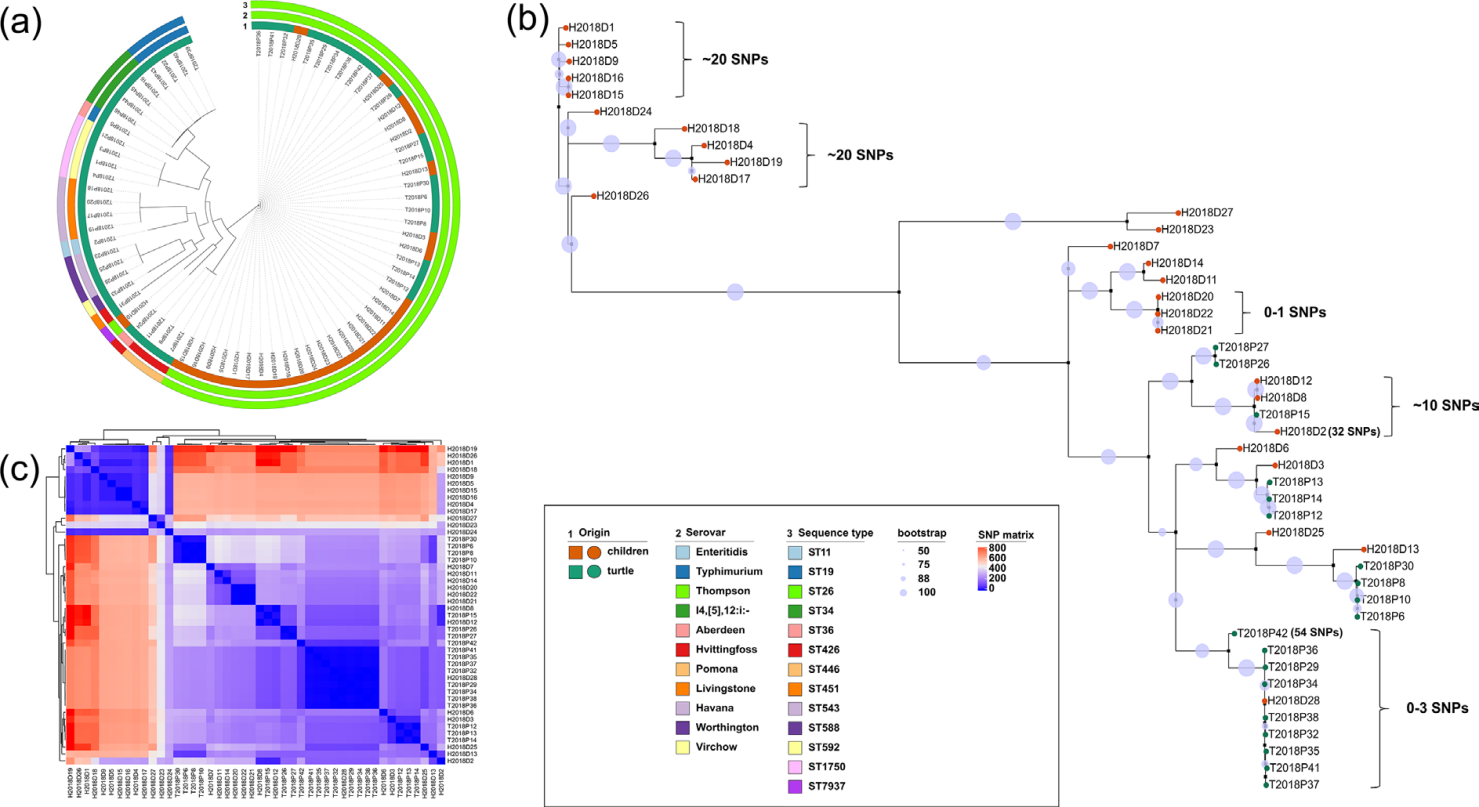
Phylogenetic tree of the whole cohort. (**a**) Maximum-likelihood tree based on 3092 core genes of the 74 *Salmonella* isolates recovered from pet turtles (*n*=46) and children with diarrhoea (*n*=28). The origins, serovars and sequence types (STs) are colour-coded in the rings from the inside out. (**b**) Maximum-likelihood tree obtained from an alignment of the SNP file generated by the CFSAN_SNP pipeline from the 46 ST26 *S*. Thompson isolates recovered from pet turtles (*n*=19) and children with diarrhoea (*n*=27) with bootstrapping (*n*=1000), using HFCDC-SM-846 (CP028729) as the reference strain. The origin from children and pet turtles is indicated as brownish orange and ocean green leaf nodes, respectively. Bootstrap values are indicated as light lavender branch labels. (**c**) SNP distance matrix between ST26 *S*. Thompson isolates generated with the CFSAN_SNP pipeline. Colours indicate pairwise SNP distances between isolates.

Twenty-eight *S*. Thompson isolates from children ≤5 years of age with diarrhoea were also collected in the enteric disease clinic of a hospital near the pet turtle sampling markets to investigate potential relationships of the dominant *Salmonella* serovar between both origins (Data Set S1 and Fig. 1). All pet turtle sampling markets are located within 5 km of the hospital. To obtain these child-associated *S*. Thompson isolates, a total of 700 faecal samples were collected from children with acute diarrhoea aged ≤5 years, during the study year. Then, all of the faecal samples were tested for *S*. Thompson using the aforementioned methods. All of the procedures performed in studies involving human participants were approved by the Research Ethics Committee of China National Center of Food Safety Risk Assessment, Beijing, China (approval no. 2014003), and written informed consents were sought from the children’s parents or guardians.

### Antimicrobial susceptibility testing

Antimicrobial susceptibility testing (AST) of the *Salmonella* spp. isolates was evaluated using the broth dilution method according to the Biofosun Gram-negative panels (Shanghai Biofosun Biotech, China) according to the manufacturer’s instructions. The minimum inhibitory concentrations (MICs) of 13 antimicrobial agents, including ampicillin (AMP), ampicillin-sulbactam (SAM), ceftazidime (CAZ), cefotaxime (CTX), imipenem (IPM), meropenem (MEM), trimethoprim-sulfamethoxazole (SXT), gentamicin (GEN), streptomycin (STR), tetracycline (TET), ciprofloxacin (CIP), nalidixic acid (NAL) and chloramphenicol (CHL), were determined, and the results were interpreted using Clinical and Laboratory Standards Institute guidelines [24]. *Escherichia coli* ATCC 25922 was used as quality control.

### DNA purification and extraction

Each isolate was grown in brain heart infusion (BHI) broth (HopeBio) at 37 °C, and genomic DNA (gDNA) was extracted and purified using an Omega EZNA Bacterial DNA kit (Omega Bio-Tek). The harvested DNA was detected by agarose gel electrophoresis and quantified by a Qubit 2.0 fluorometer (Thermo Fisher Scientific).

### Library construction and whole-genome sequencing

A total of 1 mg DNA per sample was used as input material for DNA library preparation. Sequencing libraries were generated using NEBNext Ultra DNA library prep kit for Illumina (NEB) according to the manufacturer’s recommendations, and index codes were added to attribute sequences to each sample. Briefly, the DNA sample was fragmented by sonication to a size of 350 bp, and then DNA fragments were end polished, A tailed and ligated with the full-length adaptor for Illumina sequencing with further PCR amplification. Finally, PCR products were purified (AMPure XP system), and libraries were analysed for size distribution by an Agilent 2100 Bioanalyzer and quantified using real-time PCR. The resultant DNA preparations were sequenced using an Illumina NovaSeq PE150 at Beijing Novogene Bioinformatics Technology.

### Genome assembly and analysis

Trimmomatic [25], FastQC (https://www.bioinformatics.babraham.ac.uk/projects/fastqc), SPAdes v3.14 [26] and Prokka v1.14.5 [27] were used for read quality control, assembly and annotation. MLST v2.19.0 (https://github.com/tseemann/mlst) was used for identifying the sequence type (ST). Novel STs were confirmed and assigned by uploading raw reads to the *Salmonella* database in EnteroBase (https://enterobase.warwick.ac.uk/species/index/senterica). AMR genes were mined using AMRFinderPlus v3.9.8 [28]. Virulence genes were identified with ABRicate v1.01 (https://github.com/tseemann/abricate) using the VFDB database (http://www.mgc.ac.cn/VFs/main.htm) with 95 % identity and 90 % query coverage cutoffs. *Salmonella* pathogenicity islands (SPIs) were identified with SPIFinder 2.0 (https://cge.food.dtu.dk/services/SPIFinder/) with 95 % identity and 60 % query coverage cutoffs. The mob_recon and mob_typer tools were used to identify and extract plasmid-derived contigs from the assemblies, as well as to predict the mobility, replicon and relaxase types of the identified plasmids [29]. Plasmids among the *S*. Thompson isolates shared by children and pet turtles were visualized with a Sankey diagram using an R script (v3.6.2) (https://www.r-project.org/) with the networkD3 package (https://cran.r-project.org/web/packages/networkD3/index.html). The presence/absence of AMR genes, virulence genes and plasmid replication types were used for hierarchical clustering of the genomes in the R script (v3.6.2) with the package pheatmap (https://cran.r-project.org/web/packages/pheatmap/index.html). ESBL-producing genes were compared and visualized with Easyfig v2.2.2 [30], and blast Ring Image Generator (BRIG; http://brig.sourceforge.net/).

### Core-genome and SNP phylogenetic analysis

All sequenced *Salmonella* from our study were taken as input for pan-genome analysis with core-genome alignments through Roary v3.6.0 [31]. IQ-TREE 2 [32] was then used to reconstruct the phylogenetic trees from the core-genome alignment. Meanwhile, high-quality SNPs were identified using the Center for Food Safety and Applied Nutrition and single-nucleotide polymorphism (CFSAN_SNP) pipeline (https://github.com/CFSAN-Biostatistics/snp-pipeline) with default quality filters. Specifically, the minimum base quality was 20, the minimum mapping quality was 15 and the minimum fraction of reads for SNP calls was 0.6. *S*. Thompson strain HFCDC-SM-846 (CP028729) was used as a reference genome. Plasmid and prophage regions were removed from the reference genome prior to read mapping. The SNP matrix ‘snpma_preserved.fasta’ with abnormal SNPs was used for the reconstruction of phylogenetic trees. Regions with abnormal SNP density and removal of SNPs in these regions were identified/performed by the filter_regions command of cfsan_snp_pipeline with parameters as --window_size 1000 125 15 --max_snp 3 2 1. Maximum-likelihood trees were built from the SNP alignments using IQ-TREE 2 with parameters -m MFP -b 1000 -redo [32, 33]. The phylogenetic trees were subsequently visualized through iTOL [34]. The SNPs between isolates were visualized through R script (v3.6.2) (https://www.r-project.org/) with the ComplexHeatmap package (http://bioconductor.org/packages/release/bioc/html/ComplexHeatmap.html).

### Bayesian divergence estimates

Bayesian evolutionary analysis using BEAST v1.10.4 [35] was performed to estimate node dates of *S*. Thompson strains from our study combined with publicly available WGS data. To retrieve the *S*. Thompson genomes, a core genome maximum-likelihood tree was reconstructed based on genomes from our study and 1899 *S*. Thompson genomes with isolation date and location information downloaded from the PATRIC database (https://www.patricbrc.org/). Consequently, 90 *S*. Thompson isolates clustered with the Chinese genomes were selected for the study (Data Set S1). The analysis was conducted on a core-genome alignment using snippy (https://github.com/tseemann/snippy) with *S*. Thompson strain HFCDC-SM-846 (CP028729) as a reference genome [31]. The Gubbins command of run_gubbins.py (https://github.com/nickjcroucher/gubbins) was used to remove the recombinant regions from the core genome alignment. TempEst v1.5.3 was used to check the temporal signal [36]. Marginal likelihood estimation using stepping-stone sampling was used to compare the combinations of four clock models (strict clock, uncorrelated relaxed clock, random local clock and fixed local clock), three tree priors (constant size, Bayesian skyline and birth–death process), and two substitution models [Hasegawa, Kiahino, Yano (HKY) and generalized time-reversible (GTR]). Log marginal likelihood values were in the range 20 467 456–22 467 189. The best model of GTR+gamma nucleotide substitution model, strict clock rate and constant population growth model was used. BEAST analysis was run for 80 million steps with sampling every 1000 generations. For each of the model combinations, two independent chains were performed until the effective sample size (ESS; i.e. the effective number of independent draws from the posterior distribution) for all of the parameters was greater than 200 per chain. The burn-in percentage was set to 10%, while the Bayes factors (BF) was set to BF>3 to select the best model. The target tree was selected using TreeAnnotator v1.10.4 [37] and then visualized in iTOL v4 [34].

## Results

### General characteristics of *Salmonella* isolates

A total of 46 *Salmonella* isolates were detected in pet turtles (46 of 480, 9.6 %) in 2019 in Beijing, China (Table 1). Among these 46 *Salmonella* isolates, 11 serovars were identified, with Thompson being the predominant serovar (41.3 %, 19/46), followed by Typhimurium (10.9 %, 5/46) and its monophasic variant I4[5], 12:i:- (8.7 %, 4/46), as well as Livingstone and Virchow (both 8.7 %, 4/46). Three each were identified as *S*. Havana and *S*. Hvittingfoss (6.5 %, 3/46), and one each was identified as *S*. Aberdeen, *S*. Enteritidis, *S*. Pomona and *S*. Worthington (2.2 %, 1/46). A total of 12 STs were identified among the above 46 pet turtle-associated *Salmonella* isolates, showing strong specific correlations with their serovars, as shown in Table 1 and Fig. 1. Of all 28 *S*. Thompson isolates from children with diarrhoea, 27 isolates were subtyped as ST26; the remaining one was found to be a novel ST that was subsequently confirmed and assigned ST7937 by Enterobase (https://enterobase.warwick.ac.uk/species/index/senterica).

**Table 1.** *Salmonella* isolated from pet turtles in Beijing, China

Serovars	No. of isolates, % (*n*) (*N*=480)	Sequence type
Children with diarrhoea		
Thompson	−	ST26 (27), ST7937 (1)
Turtle		
Thompson	4.0 (19)	ST26 (19)
Typhimurium	1.0 (5)	ST19 (4), ST36 (1)
I4 [5],12:i:-	0.8 (4)	ST34 (4)
Livingstone	0.8 (4)	ST543 (4)
Virchow	0.8 (4)	ST1750 (4)
Havana	0.6 (3)	ST588 (3)
Hvittingfoss	0.6 (3)	ST446 (3)
Aberdeen	0.2 (1)	ST426 (1)
Enteritidis	0.2 (1)	ST11 (1)
Pomona	0.2 (1)	ST451 (1)
Worthington	0.2 (1)	ST592 (1)
Total	9.6 (46)	−

### Antimicrobial susceptibility testing of *Salmonella* isolates from pet turtles and children with diarrhea

The AST results showed that all the 74 *Salmonella* isolates (46 from pet turtles and 28 from children with diarrhoea) were susceptible to both imipenem and meropenem (Table 2 and Fig. S1). Of 46 pet turtle-associated isolates, 91.3 % were resistant to at least one antimicrobial. All isolates of the serovars Thompson, Typhimurium (except the one ST36 isolate), I4 [5],12:i:-, Livingstone, Virchow and Hvittingfoss from pet turtles were MDR, whereas isolates of the serovars Aberdeen, Havana, Worthington and Enteritidis were pan-susceptible (Table 2 and Fig. S1). The pet turtle-associated *S*. Thompson isolates also showed higher MDR (89.5 %, 17/19) than those from children with diarrhoea (57.1 %, 16/28) (*P*<0.05). Most (60.7 %, 28/46) of pet turtle-associated *Salmonella* isolates demonstrated R-type ASSuT, including 16 *S*. Thompson and all of the I4[5],12:i:-, Livingstone and Virchow serovar isolates. The rate of R-type ASSuT among pet turtle-associated *S*. Thompson (84.2 %, 16/19) was higher than that among children with diarrhoea (28.6 %, 8/28) (*P*<0.01).

**Table 2.** Antimicrobial resistance of *Salmonella* recovered from pet turtles and children with diarrhoea

Antimicrobials	Turtle, % (*n*)		Children, % (*n*)	Pearson chi-square test *P* value
	Total *N*=46	Thompson *N*=19	Thompson *N*=28	
R-type ASSuT	60.7 (28)	84.2 (16)	28.6 (8)	<0.01
AMR resistance (≥1 class of antimicrobial)	91.3 (42)	100 (19)	71.4 (20)	<0.05
MDR (≥3 classes of antimicrobials)	80.4 (37)	89.5 (17)	57.1 (16)	<0.05
MDR (≥5 classes of antimicrobials)	67.4 (31)	84.2 (16)	39.3 (11)	<0.01

### Phylogenetic characteristics and relatedness among *S*. Thompson from pet turtles and children with diarrhea

The phylogenetic tree for all of the 74 *Salmonella* isolates in our study was reconstructed with a set of 3092 concatenated core genes (Fig. 1a). Isolates of the same serovar were clustered within the same clade with one exception in that the novel ST7937 *S*. Thompson isolate was clustered with the *S*. Aberdeen isolate (ST426, *n*=1). The *S*. Typhimurium isolates (ST19, *n*=4; ST36, *n*=1) and its monophasic variant I4.[5],12:i:- isolates (ST34, *n*=4) were further separated by their STs. The 46 ST26 *S*. Thompson isolates from pet turtles (*n*=19) and children with diarrhoea (*n*=27) formed a cluster distinct from those of other serovars.

To determine the genomic relatedness of these 46 ST26 *S*. Thompson isolates, SNP-based phylogenies were constructed using the CFSAN_SNP pipeline (Fig. 1b). The median [interquartile range (IQR) and range] SNP differences between all of the 46 ST26 *S*. Thompson isolates were 274 (143, 606 and 0, 996) (Fig. 1c). Of note, 8 of the 28 child-associated isolates were found to be mixed with the pet turtle-associated isolates and showed very low SNP differences with bootstrap support >90 (Fig. 1b). In detail, one child-associated isolate (H2018D28) was clustered with nine pet turtle-associated isolates with as few as 0–3 SNP differences. One pet turtle-associated isolate (T2018P15) was found to be clustered with three child-associated isolates with ~10–32 SNP differences.

### Bayesian analysis of the ST26 *S*. Thompson genomes from pet turtles and children with diarrhoea

**Fig. 2.**
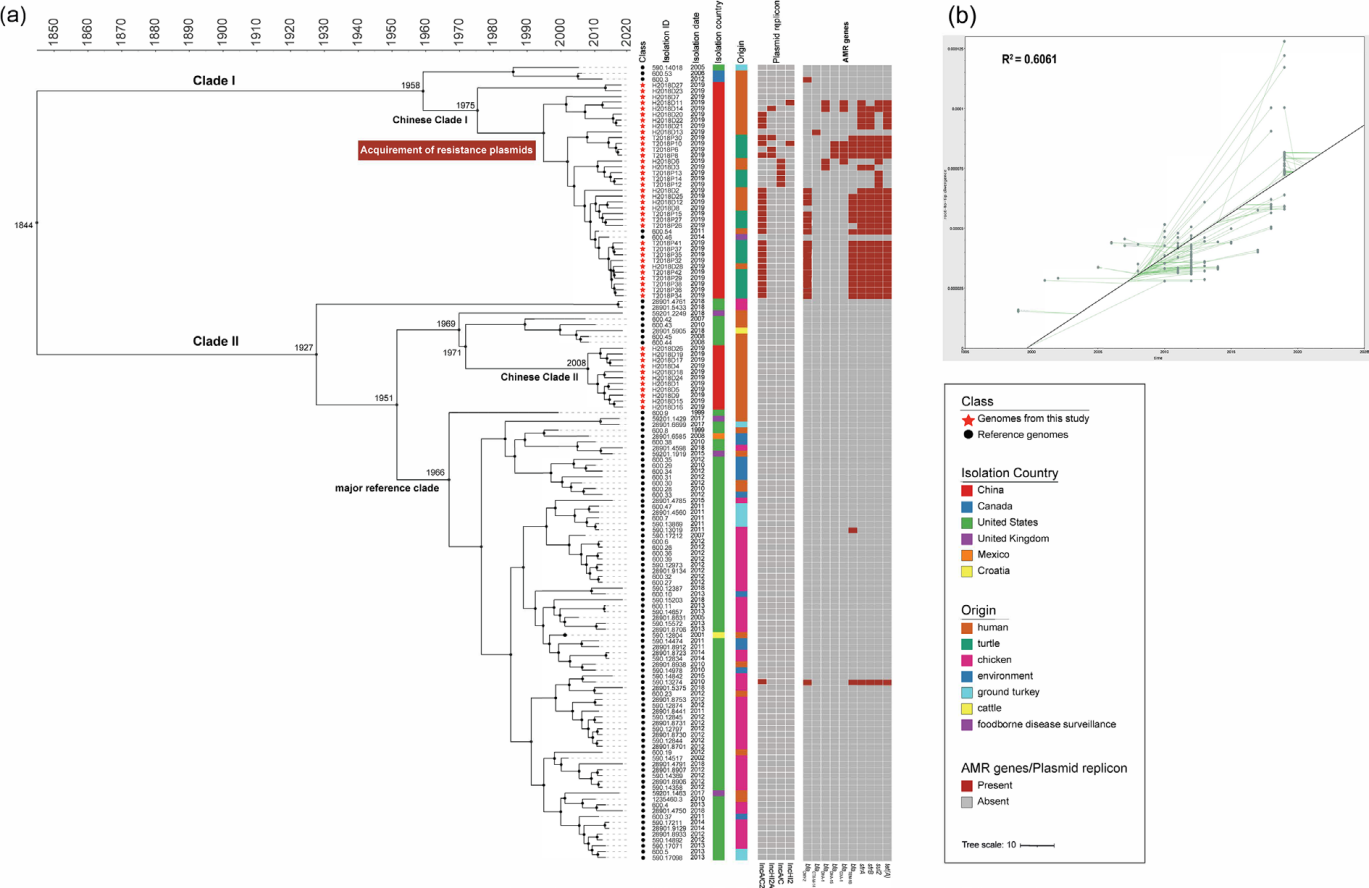
Bayesian phylogenetic analysis of 136 ST26 *S*. Thompson genomes including 46 genomes from our study and 90 reference genomes. (**a**) Isolates from our study and reference isolates are indicated as red star and black circle nodes, respectively. Colours in columns illustrate countries and origins. Heatmaps shows the presence (brick) or absence (grey) of major plasmid replicon types or AMR genes, respectively. (**b**) Root-to-tip plot generated by TempEst to show the regression of genetic distance against sampling time.

A Bayesian phylogenetic tree was developed to decipher the evolutionary history of 136 ST26 *S*. Thompson isolates (46 from our study and 90 from PATRIC) and to compare the genomic features between the Chinese isolates and those from other countries (Fig. 2a). The pan-genome analysis identified 3996 core genes across all 136 of the ST26 *S*. Thompson genomes. The *R* ^2^ (0.6) coefficient indicated a correlation between the isolation date and sequence divergence, which indicated that our data set was suitable for temporal analysis (Fig. 2b). The evolutionary rate for the 136 isolate clade was predicted to be 5.48×10^−7^ substitutions/site/year [95 % highest posterior density (HPD)=4.64×10^−7^ to 6.78×10^−7^].

All 136 of the *S*. Thompson isolates shared a most recent common ancestor (MRCA) that existed circa 1844 (95 % HPD, 1788–1901). Resembling what was observed in the maximum-likelihood phylogeny, these 136 genomes were divided into two major ancestral clades (Clades I and II) that originated circa 1958 (95 % HPD, 1937–1981) and 1927 (95 % HPD, 1902–1949), respectively. The 48 Chinese genomes (46 from this study and two previously reported) formed two separate clades among the genomes of both ancestral clades. Chinese Clade I (*n*=37) originated circa 1975 (95 % HPD, 1962–1987) and most isolates (33/37) in this clade were found to possess resistance plasmids that carried multiple AMR genes. All of the isolates of Chinese Clade II (*n*=11), originating circa 2008 (95 % HPD, 1999–2018), were pan-susceptible (carrying no resistant genes). Both Chinese clades belonged to distinct lineages with isolates from European or American countries. For instance, the major reference clade (*n*=72) originating circa 1966 (95 % HPD, 1951–1980) was much earlier than the Chinese Clade II. Furthermore, more AMR genes, especially plasmid-mediated, were found from *S*. Thompson isolates within Chinese Clade I than in other clades. For instance, nearly all the IncA/C and IncHI plasmids were identified from isolates within this clade. Similarly, *S*. Thompson isolates within Chinese Clade I carried most of the AMR genes, such as genes conferring resistance to the β-lactam antimicrobials (*bla*_CMY-2_, *bla*_CTX-M-14_, *bla*_DHA-1_, *bla*_DHA-15_, *bla*_OXA-1_ and *bla*_TEM-1B_), and the R-type ASSuT [*bla*_TEM-1B_, *strAB*, *sul2* and *tet(A*)]. By contrast, isolates from other clades rarely carried resistance plasmids and AMR genes.

### Antimicrobial resistance genes in *Salmonella* genomes

In total, 42 acquired AMR genes were detected (Fig. S1 and Data Set S1), along with a single mutation in the quinolone resistance-determining regions (QRDRs) gene *gyrA* and two quaternary ammonium-resistant genes (*qacEdelta1* and *qacL*). Five types of acquired AMR genes were detected in more than half of pet turtle-associated *Salmonella* isolates. These included *sul1* (*n*=24) and *sul2* (*n*=28, encoding resistance to sulphonamide), *bla*_TEM-1B_ (*n*=24, ampicillin), *tet(A*) (*n*=24, tetracycline), *strA* (*n*=23) and *strB* (*n*=23, streptomycin), along with *floR* (*n*=23, phenicol). Five β-lactam AMR genes were detected in 32 pet turtle-associated *Salmonella* isolates, with *bla*_TEM-1B_ (*n*=24) the most frequently detected, followed by *bla*_CMY-2_ (*n*=12), *bla*_OXA-1_ (*n*=11), *bla*_DHA-15_ (*n*=3) and *bla*_OXA-10_ (*n*=1). Among these, the *bla*_CMY-2_ and *bla*_DHA-15_ genes were AmpC β-lactamases, while the *bla*_OXA-10_ gene was an ESBL. For fluoroquinolone resistance, four single-point mutations were detected in *gyrA* of the pet turtle-associated *Salmonella* isolates (resulting in the following amino acid substitutions: S83Y, *n*=4; D87G, D87N and S83F, each *n*=1) along with nine plasmid-mediated quinolone resistance (PMQR) genes, including *aac(6’)-Ib-cr* (*n*=16), *qnrS1* (*n*=14), *qepA8* (*n*=4), *qnrB6* (*n*=4), *qnrB4* (*n*=3), *qnrS2* (*n*=3), *qnrA1* (*n*=2), *oqxA* and *oqxB* (*n*=1). Additionally, other AMR genes encoding resistance to aminoglycosides, chloramphenicol, trimethoprim, sulphonamide, tetracycline, bleomycin, macrolide and rifampin were also detected.

All of the isolates (*n*=38) of the serovars Thompson, Typhimurium (except the one ST36 isolate), I4 [5],12:i:-, Livingstone, Virchow and Hvittingfoss from pet turtles were found to harbour genes encoding MDR, which matched the MDR phenotypes. Moreover, among the 28 R-type ASSuT, only three isolates harboured the typical resistant genes profile *bla*_TEM-1B_-*strAB-sul2-tet(B*), all of which were *S*. I4 [5],12:i:-; while 16 isolates (15 *S*. Thompson and one *S*. Virchow) harboured the resistant genes profile *bla*_TEM-1B_-*strAB-sul2-tet(A*).

The matching between AMR genotype and phenotype was analysed among the 74 studied *Salmonella* isolates (Table S1). Of the resistant isolates, more than 83.3 % (83.3–97.8 %) were subsequently found to carry related AMR genes. Meanwhile, more than 81.4 % 81.4–100 %) of isolates positive for AMR genes showed resistance to related antimicrobials.

### Resistance plasmids and acquired AMR genes shared between pet turtle- and child-associated *S*. Thompson isolates

In total, 27 types of plasmids were identified from 59 *Salmonella* genomes in this study, among which 26 types of plasmids from 52 *Salmonella* genomes harboured AMR genes. Of these 26 types of resistant plasmids, 11 could be assigned the replicon types, and 15 were non-typeable and were described with their names in the database of MOB_suite (Fig. S1 and Data Set S1). Of the pet turtle-associated *Salmonella* strains, 11 types could be assigned the replicon types, with the IncA/C2 (*n*=15) plasmids being the most detected, all identified in *S*. Thompson (Fig. S2A). All the IncA/C2 (*n*=15), IncA/C (*n*=6), IncHI1A (*n*=8), IncHI2 (*n*=3), IncI1 (*n*=1) and IncX2 (*n*=1) plasmids, as well as most IncHI2A (5/6) plasmids were predicted to be conjugative plasmids. Col440I (*n*=1), IncQ2 (*n*=3) and several non-typeable plasmids were predicted to be mobilizable, whereas other plasmids were predicted to be non-mobilizable.

Thirty-seven acquired AMR genes were found among the pet turtle-associated *Salmonella* genomes. These 37 AMR genes were most frequently identified from *S*. Thompson (25/37), followed by *S*. Virchow (16/37), *S*. I4 [5],12:i:- (16/37) and *S*. Typhimurium (14/37) (Fig. S2B and C). Similarly, several AMR genes could be carried on multiple plasmids (Fig. S2D). Furthermore, 18 AMR genes were identified to co-occur with the IncHI2A plasmid replicon type.

**Fig. 3.**
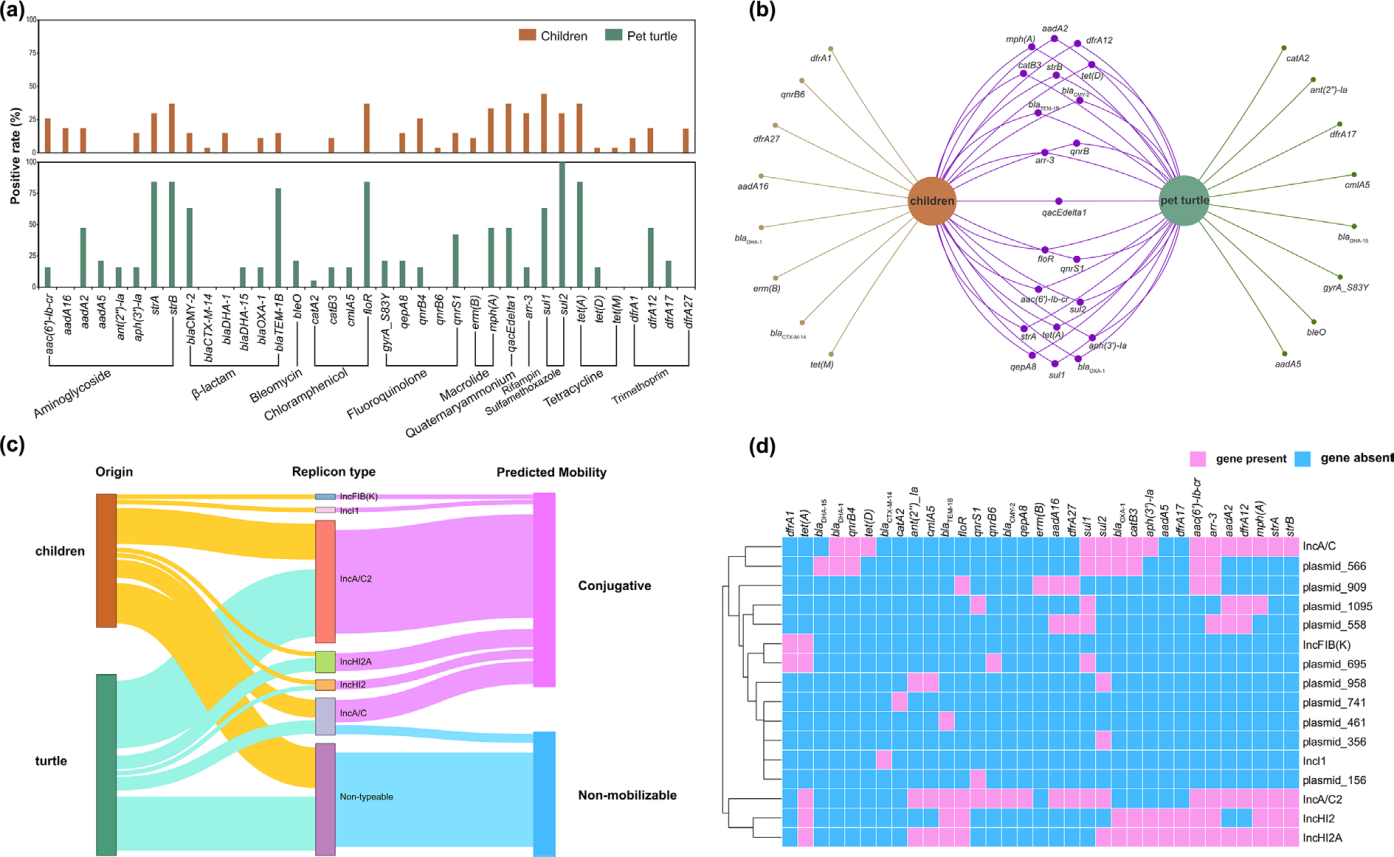
(**a**) Positive rate of diverse AMR genes among pet turtle- and child-associated *S*. Thompson isolates in this study. (**b**) AMR genes shared across host species (pet turtles and children). (**c**) Sankey diagram exhibiting the origins of isolates, replicon types and predicted mobility of potential resistant plasmids detected from pet turtle- and child-associated *S*. Thompson isolates in this study. (**d**) Heatmap of presence (lavender pink) or absence (sky blue) of AMR genes identified from pet turtle- and child-associated *S*. Thompson isolates co-occurring with different plasmid replicon types in this study.

Twenty-eight acquired AMR genes were identified from the genomes of child-associated *S*. Thompson isolates, among which 21 were shared with the pet turtle-associated *S*. Thompson isolates despite different positive rates (Fig. 3a, b). The child-associated *S*. Thompson isolates carried 16 types of plasmids, including six typeable and 10 non-typeable plasmids (Fig. 3c, d). IncA/C2 (*n*=23) plasmids were the most frequently detected, followed by IncA/C (*n*=5), IncHI2A (*n*=4) and IncHI2 (*n*=2), all of which were shared with pet turtle-associated *S*. Thompson isolates and predicted to be conjugative plasmids (except two non-mobilizable IncA/C plasmids).

### Genetic environment of β-lactam AMR genes in *Salmonella* genomes

**Fig. 4.**
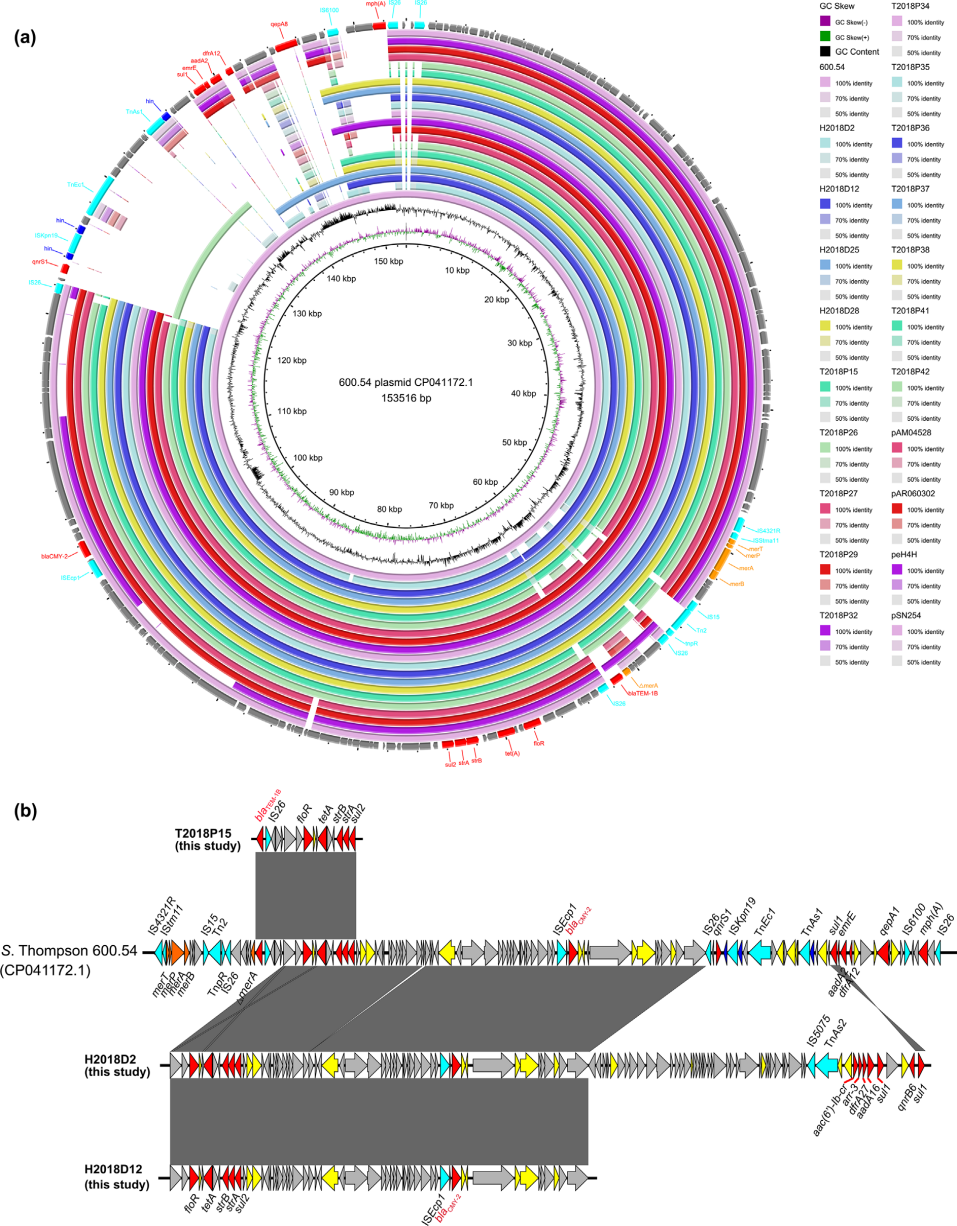
Comparison (**a**) and genetic structure (**b**) of the CMY-2 and TEM-1B genes in this study and those in the online NCBI database. Contigs potentially plasmid-derived and reference plasmids are colour-coded in the rings from inside out. Transposons/insertion sequences, recombinase genes, resistant genes, hypothetical genes, heavy metal resistant genes, functional genes and phage shock proteins are colour-coded and labelled in the outmost ring indicating the reference plasmids used for comparison.

The AmpC β-lactamase-encoding gene *bla*_CMY-2_ (16 isolates including 12 from pet turtles and four from children) were linked to the transposable element IS*Ecp1* upstream and co-occurred with the IncA/C2 plasmid replicon types in the *S*. Thompson isolates (Fig. 4a, b, and Data Set S1). In detail, one resistant gene cassette *floR-tet(A)-strB-strA-sul2* ~36 kb upstream of the *bla*_CMY-2_ gene was detected, with or without the *bla*_TEM-1B_ (*n*=14) gene flanked by the transposable element IS*26*. Upstream of this resistant cluster, one mercury-resistant operon (*merT-merP-merA-merB*) was found to be flanked by several insertion sequences (IS*4321R*, IS*Stma11*, IS*15* and IS*26*) and transposons (Tn*2* and tn*pR*). This arrangement was similar to a resistant *S*. Thompson plasmid (CP041172.1) previously recovered from a clinical human patient in China in 2011, which also belonged to the Chinese Clade I (Figs 2a and 4). Furthermore, these IncA/C2-mediated arrangements were also similar to four previously described IncA/C2 plasmids found in two *E. coli* (plasmids pAR060302 and peH4H) and two *Salmonella* Newport strains (plasmids pAM04528 and pSN254a) (Fig. 4). However, the ST26 *S*. Thompson isolates in this study did not carry the resistance cassette *sul1-ermE-aadA2-dfrA12-mphA* as found in the reference plasmids. One child-associated *S*. Thompson isolate (H2018D2) carried an extra AMR gene cassette *aac(6’)-Ib-cr-arr-3-dfrA27-aadA16-sul1-qnrB-sul16*, linked to IS*5075* and Tn*As2* (Fig. 4b).

**Fig. 5.**
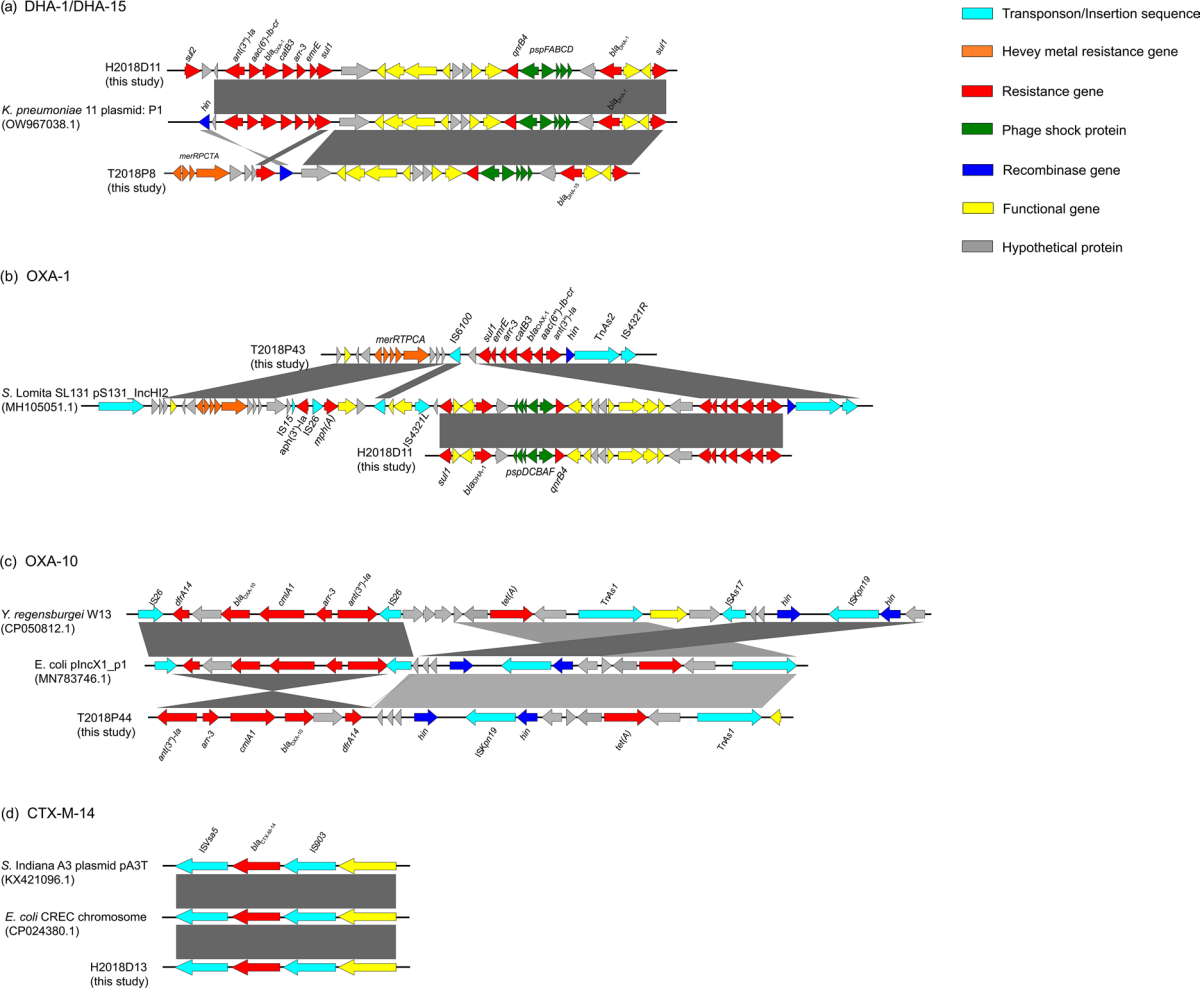
Genetic structure of potentially plasmid-mediated *bla*_DHA-1_ and *bla*_DHA-15_ (**a**), *bla*_OXA-1_ (**b**), *bla*_OXA-10_ (**c**) and *bla*_CTX-M-14_ (**d**) genes in this study. Transposons/Insertion sequences, recombinase genes, resistant genes, hypothetical genes, heavy metal resistant genes, functional genes and phage shock proteins are colour-coded.

Another two AmpC β-lactamase-encoding genes, *bla*_DHA-1_ gene from child-associated *S*. Thompson (*n*=4) and *bla*_DHA-15_ gene from pet turtle-associated *S*. Thompson (*n*=3), were found to have only one amino acid mutation (D165Y) and were located in the same non-mobilizable plasmid (plasmid_566), sharing similar genetic structure as that of the plasmid of *Klebsiella pneumoniae* isolate 11 (OW967038.1) (Figs 5a, S3A and Data Set S1). Moreover, two extra AMR genes *sul1* and *qnrB4* were also detected near these two AmpC β-lactamase-encoding genes, comprising a resistant gene cassette (*qnrB4-bla*_DHA-1/15_-*sul1*). A phage shock protein (*psp*) operon (*pspF-pspA-pspB-pspC-pspD*) was found to insert into this resistant cassette. One child-associated *S*. Thompson isolate (H2018D11) carried an extra resistant cassette *sul2-ant(3’’)-Ia-aac(6’’)-Ib-cr-catB3-blaOXA-1-arr-3-emrE*. Besides, the genetic environment of two other ESBLs, OXA-10 and CTX-M-14, as well as one non-ESBL OXA-1, were also analysed (Figs 5b, d and S3B, D).

### Distribution of virulence-associated genes

Among isolates in this study, the types of virulence genes were similar and the number of virulence factors ranged between 99 and 113 (Data Set S1). Nearly all of the *Salmonella* isolates contained multiple virulence genes including those for adherence (*fimC/D/F/H/I*, *misL, ratB, shdA* and *sinH*) and invasion (*invA/B/C/E/F/G/H/I/J* genes). Analysis of SPIs showed that the types of SPIs in *Salmonella* of the same serovar were consistent in this study. No difference was found between *S*. Thompson isolates from pet turtles and children with diarrhoea in SPIs. Compared with *S*. Thompson, other major detected serovars, such as the *S*. Havana isolates (*n*=3), lacked SPI-13, SPI-14 and CS54_island but carried SPI-8; the *S*. Livingstone isolates (*n*=4) lacked SPI-13, SPI-14 and C63PI; and four of five *S*. Typhimurium isolates and all four *S*. Virchow isolates lacked C63PI. Of note, several virulence genes were also found to be linked to specific serovars; for instance, the adherence genes *lpf* (*lpfA*, *lpfB*, *lpfC*, *lpfD*,and *lpfE*) were present in all of the isolates but absent in isolates of the serovars Livingstone and Pomona.

## Discussion

Turtles are considered a well-known reservoir of a wide variety of *Salmonella* species, representing numerous subspecies and serovars and can be easily colonized with vertical and horizontal transfer and shed pathogens intermittently [18]. Many reports involved turtle-associated salmonellosis in children under 5 years of age, and contact with turtles is considered to have a particularly high risk of infection [19, 20]. Worryingly, the emergence of MDR, especially ESBL-producing *Salmonella* is increasing worldwide, including in China [19, 21, 38]. However, few data are available on the prevalence and genomics characteristic of MDR and β-lactamase-producing *Salmonella* among pet turtles in China. In this study, we surveyed the prevalence, serovar distribution, MDR profiles, β-lactamase-encoding genes, genomic context and Bayesian divergence of *Salmonella* isolates collected from pet turtles in Beijing, China, during 2019, together with *S*. Thompson genomes from children with diarrhoea and other countries.

According to previous reports, the rates of *Salmonella* detection among pet turtles ranges from 14.5 to 60 % [9, 21, 39]. Overall, 46 *Salmonella* isolates (9.6 %) were recovered from 480 pet turtles in our study, supporting the suggestion that pet turtles have become a considerable reservoir of *Salmonella*. Moreover, the 11 serovars identified in this study were also frequently reported in turtles in previous studies [16, 40], representing potential public health risks. Therefore, recognition of the hazards associated with pet turtles is critical to avoid negative consequences for human health.

Notably, *S*. Thompson was the predominant serovar among all of the 46 pet turtle-associated isolates. This observation is in accordance with those reported from turtles in Spain (59.3 %) and from other studies in China (five provinces, 45.2 %; Shanghai, 19.4 %), but different from reports from the West Indies, where the dominant serovar is *S*. Montevideo [9, 12, 21, 41]. Furthermore, nearly all of the *S*. Thompson isolates from pet turtles and children with diarrhoea in this study were identified as ST26, which is also the predominant ST of *S*. Thompson in human-, animal- and food-associated samples in previous studies [9, 21, 42]. Some of the serovars identified here have also been linked to human salmonellosis, such as *S*. Pomona, which is considered to be particularly pathogenic, causing approximately 18 % of human salmonellosis cases due to turtle exposure in the USA between 2006 and 2014 [43]. Therefore, genetic relationships between isolates from different sources should be clarified to determine potential transmission. Close genetic relatedness between isolates from different sources may indicate potential spread among humans, animals and in their associated environments.

Herein, we found that pet turtle-associated *Salmonella* isolates showed a relatively high MDR (80.4 %) phenotype, with resistance to tetracycline (91.3 %) being the most detected, consistent with the fact that this antimicrobial is commonly used in the context of animal production in China [44]. In contrast, isolates from pet turtles in Korea and Spain were pan-susceptible [45, 46], which might be due to different antimicrobial usage (AMU) policies between countries. These findings are important as any *Salmonella* strains showing resistance to these drugs are a major concern for public health [47]. In this study, 80.4 % of the pet turtle-associated isolates identified as *Salmonella* serovars Thompson, Typhimurium (except the one ST36 isolate), I4[5],12:i:-, Livingstone, Virchow and Hvittingfoss from pet turtles were MDR, which is consistent with previous findings obtained in China [9, 21]. Moreover, previous studies have shown that the *S*. I4[5],12:i:- strains are often resistant to ampicillin, streptomycin, sulfonamides and tetracycline (R-type ASSuT) [48, 49]. In total, 60.7 % of pet turtle-associated *Salmonella* isolates in this study were ASSuT-resistant, including not only *S*. I4[5],12:i:- isolates (*n*=4) but also 84.2 % of *S*. Thompson isolates, and all of the *S*. Livingstone and *S*. Virchow isolates. Therefore, these resistant pet turtle-associated *Salmonella* isolates might cause severe infections and limit the choice of antimicrobial usage in clinical therapy.

The phylogenetic tree including all of the isolates from pet turtles and children with diarrhoea showed that *Salmonella* isolates exhibited strong clustering by serovars and STs, which is consistent with previous studies [21, 50]. The phylogenetic distance between the ST26 *S*. Thompson and other *Salmonella* serovars was relatively long, which has also been observed in recent research [51]. According to consensus on the use of WGS data for pathogens built by the FDA, fewer than 20 SNPs support a strong match and a bootstrap value of more than 0.90 supports a monophyletic relationship [52]. In this study, two sub-clades including pet turtle- and child-associated *S*. Thompson isolates were found to have 0–10 SNPs with bootstrap support higher than 0.90, suggesting that these isolates might arise from the same source and that pet turtles could serve as important reservoirs of infections for children.

Bayesian analysis of *S*. Thompson genomes identified two major clades with their emergence from estimated origins around 1844 and divergence occurring around 1927, which is consistent with the first discovery of this serovar in 1924 from an infected patient in the UK [16]. The Chinese lineage (Chinese Clades I and II) was shown to have a more recent evolutionary history (after 1975) and greater diversification (around 2000) compared with those from other countries, which is in line with the fact that large-scale turtle and tortoise farms were established in China during the 1980s [53], and the number of new commercial turtle farms increased steadily after the late 1990s [54]. Bayesian analysis also revealed that most ST26 *S*. Thompson isolates from both pet turtles and children with diarrhoea within the Chinese Clade I carried multiple AMR genes potentially plasmid-mediated. This might explain the main divergence in the Chinese *S*. Thompson isolates around 1995. A similar observation was also found among *S*. Typhimurium strains in a recent Chinese report [55], potentially driven by increased economic growth and antimicrobial usage in China [56]. Our findings suggest a specific clonal establishment of Chinese ST26 *S*. Thompson, particularly carrying AMR genes potentially plasmid-mediated and having the potential to spread between children and pet turtles.

Few, if any, data on ESBL- and AmpC β-lactamase-producing *Salmonella* isolates in pet turtles are currently available. In 2022, a study on *Salmonella* from turtles for human consumption in Hong Kong exhibited relatively high detection rates of AMR genes (61.9 % resistant to at least one antimicrobial, 33.3 % harbouring multiple AMR genes and 23.8 % being ESBLs), with *S*. Thompson, *S*. Manhattan and *S*. IIIb 50:k:z the most frequently detected [57]. In this study, the *S*. Thompson, *S*. Typhimurium (including *S*. I4[5],12:i:-). and *S*. Virchow isolates were found to possess the most AMR genes, which is different to those in Hong Kong [57]. Besides, the ESBL-producing gene *bla*_OXA-10_ and AmpC β-lactamase-encoding genes *bla*_CMY-2_ and *bla*_DHA-15_, as well as other AMR genes conferring resistance to aminoglycosides, chloramphenicol, fluoroquinolones, sulfonamides, tetracyclines and trimethoprim, were detected in pet turtle-associated *Salmonella* in this study. Our findings indicated that these antimicrobials might be widely misused and overused in pet turtle breeding or clinical veterinary practice, leading to serious resistance in pet turtles and consequent infections in humans.

Two dominant Inc group plasmid replicon types, IncA/C (IncA/C and IncA/C2) and IncHI (IncHI1A and IncHI2/2A), identified in this study were found to co-occur with multiple AMR genes, especially the main detected gene *bla*_CMY-2_, which has been reported in human, animal and food sources worldwide [58]. Furthermore, genomic analysis showed that the *bla*_CMY-2_ (*n*=16) and *bla*_TEM-1B_ (*n*=14) genes mostly coexisted with the IncA/C2 plasmid replicon types in the pet turtle- and child-associated *S*. Thompson isolates. IncA/C2 plasmids are important conjugative vectors for crucial resistant genes, especially CMY and TEM, among members of the family *Enterobacteriaceae* [59, 60]. Moreover, the arrangement of these cassettes was similar to that found in an MDR *S*. Thompson plasmid (CP041172.1) from a clinic in China during 2011. This finding suggested that these IncA/C2 plasmids might have been widespread in *S*. Thompson among pet turtles and humans in China.

To date, only one *S*. Thompson isolate having the *bla*_DHA-1_ gene was recovered from a paediatric inpatient with diarrhoea from Jiangxi, China, which was first isolated from an *S*. Enteritidis strain in Saudi Arabia [61, 62]. In the current study, the *bla*_DHA-1_ gene was found in four child-associated *S*. Thompson isolates. Although no pet turtle-associated *S*. Thompson isolates were positive for the *bla*_DHA-1_ gene, the *bla*_DHA-15_ gene, a variant of *bla*_DHA-1_ (D165Y), was detected from pet turtle-associated *S*. Thompson isolates. Notably, the *bla*_DHA-15_ gene was frequently detected in *K. pneumoniae* [63], whereas no data are available for *Salmonella*. To the best of our knowledge, this was the first detection of *bla*_DHA-15_ from *Salmonella* strains. Our findings expand the knowledge regarding the prevalence of DHA-1/15 AmpC β-lactamase-producing *Salmonella* among children with diarrhoea and pet turtles. The *bla*_OXA-1_ gene (*n*=14), along with other AMR genes identified from pet turtle- and child-associated *Salmonella* isolates, mainly co-occurred with the IncHI2 (*n*=9) plasmid replicon type in this study. Notably, acquisition and dissemination of AMR genes in IncHI2 plasmids can resemble a nested Russian doll [64], encoding resistance to the most critically important antimicrobials and posing an enormous challenge to public health.

Several fimbrial genes (*bcf*, *fim*, *inv*, *csg*) and type III secretion systems 1 and 2, implicated in cell invasion and viability of the bacteria within phagocytes, were common to all of the *Salmonella* isolates. These genes are probably part of the core genes with an essential function for *Salmonella* serovars [50]. SPIs, especially the important SPI-1 to SPI-5, were detected in all of the *Salmonella* strains in this study, which are reported to encode the type III secretion system, the killing effect of bacterial escape from macrophages, the survival of *Salmonella* in host macrophages, a type I secretion system, intestinal mucosal fluid secretion and the inflammatory response [65, 66]. Our data showed that *S*. Thompson isolates recovered from pet turtles contained the same virulence genes as those from children with diarrhoea.

## Conclusion

In the present study, we demonstrate the important role of pet turtles as a reservoir for *Salmonella*, representing a variety of serovars and MDR strains. These MDR pet turtle-associated *Salmonella* isolates could pose potential hazards to humans, particularly children, as a susceptible population. The dominant ST26 *S*. Thompson isolates among pet turtles comprised clusters that were phylogenetically close to those from children with diarrhoea (<20 SNP differences with each other), providing evidence of potential interspecies transmission. Bayesian analysis indicated that the Chinese ST26 *S*. Thompson strains exhibited a more recent evolutionary history than the European and American ones; these subsequently evolved along two distinct pathways resulting in one susceptible clade and one MDR clade. Our data indicate the necessity for a One Health approach to effectively monitor the spread of resistance. Dedicated disinfection procedures and effective legislation should be formulated to maintain a *Salmonella*-free status among pet turtles from farm production to placement in the home. Because our study only provided evidence to show the close genetic relatedness of *S*. Thompson between children and pet turtles, further research is urgently needed to clarify the transmission between these hosts.

## Supplementary Data

Supplementary material 1

Supplementary material 2
