# Supplementary material for: Emergence and genomic characteristics of multi-drug-resistant Salmonella in pet turtles and children with diarrhoea
Source: Microb Genom. 2024 Jan 3;10(1):001164. doi: 10.1099/mgen.0.001164 (PMC10868623; doi:10.1099/mgen.0.001164)
Supplement: Supplementary material 1 [file mgen-10-1164-s001.pdf]

Table S1 The matching between AMR genotype and phenotype of 74 *Salmonella* isolates in this study.

Antimicrobials	Isolates showing AMR phenotypes, n	AMR genes, n (%)	Isolates positive with AMR genes, n	Resistant isolates, n (%)
Aminoglycosides	43	41 (95.3)	44	41 (93.2)
β-lactam	45	42 (93.3)	42	42 (100)
Phenicols	45	41 (91.1)	44	41 (93.2)
Fluoroquinolones	42	35 (83.3)	43	35 (81.4)
Folate Pathway Inhibitors	46	45 (97.8)	51	45 (88.2)
Tetracyclines	52	49 (94.2)	49	49 (100)

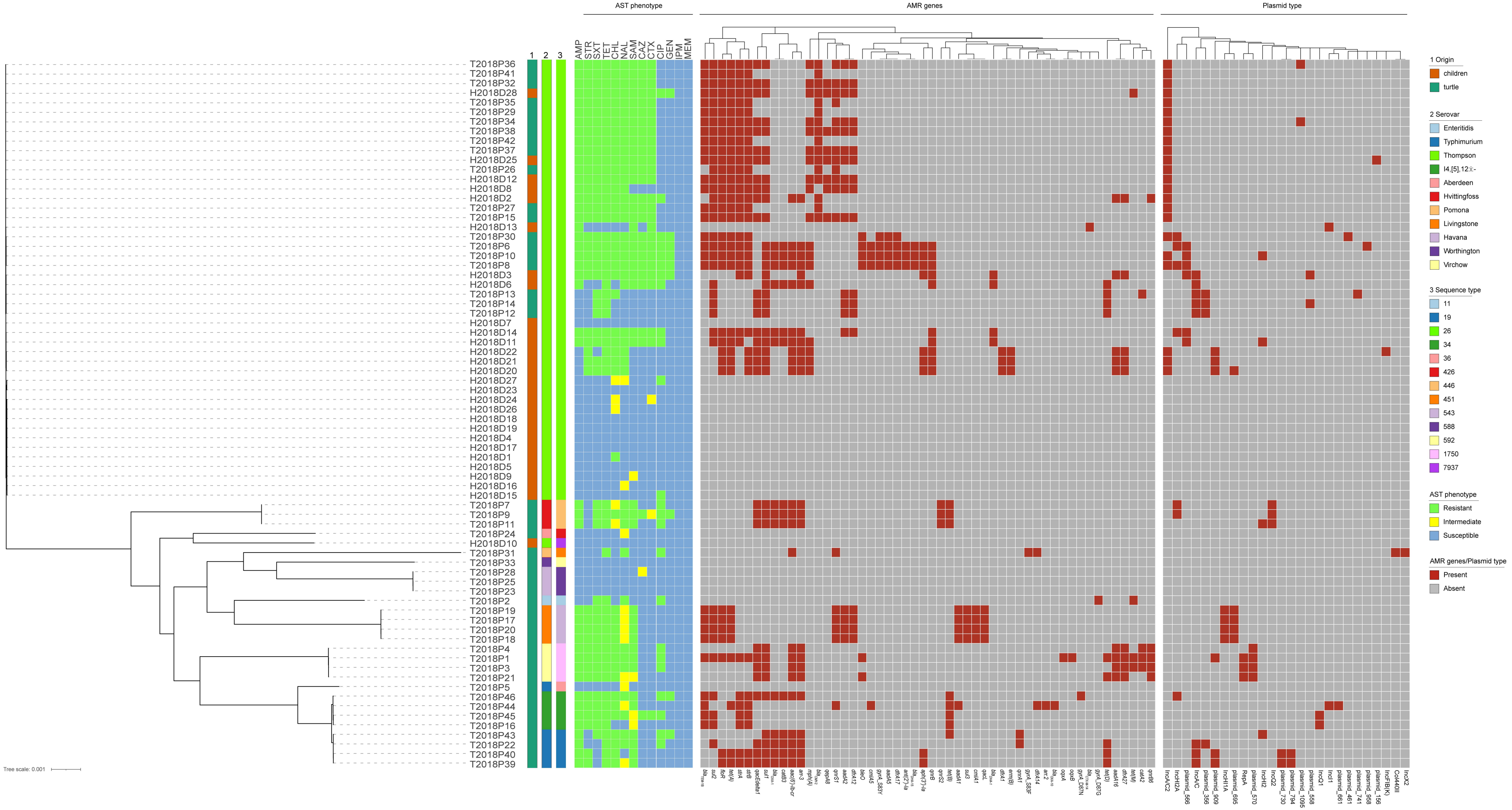

Figure S1. Maximum-likelihood tree based on 3,092 core genes of the 74 *Salmonella* isolates recovered from pet turtles (n = 46) and children with diarrhea (n = 28). Colors in columns illustrate origins, serovars, and sequence types, respectively. Heatmaps exhibit the resistant (light glass green), intermediate (lemon), and susceptible AST phenotypes, and presence (brick) or absence (grey) of AMR genes or plasmid replicon types, respectively.

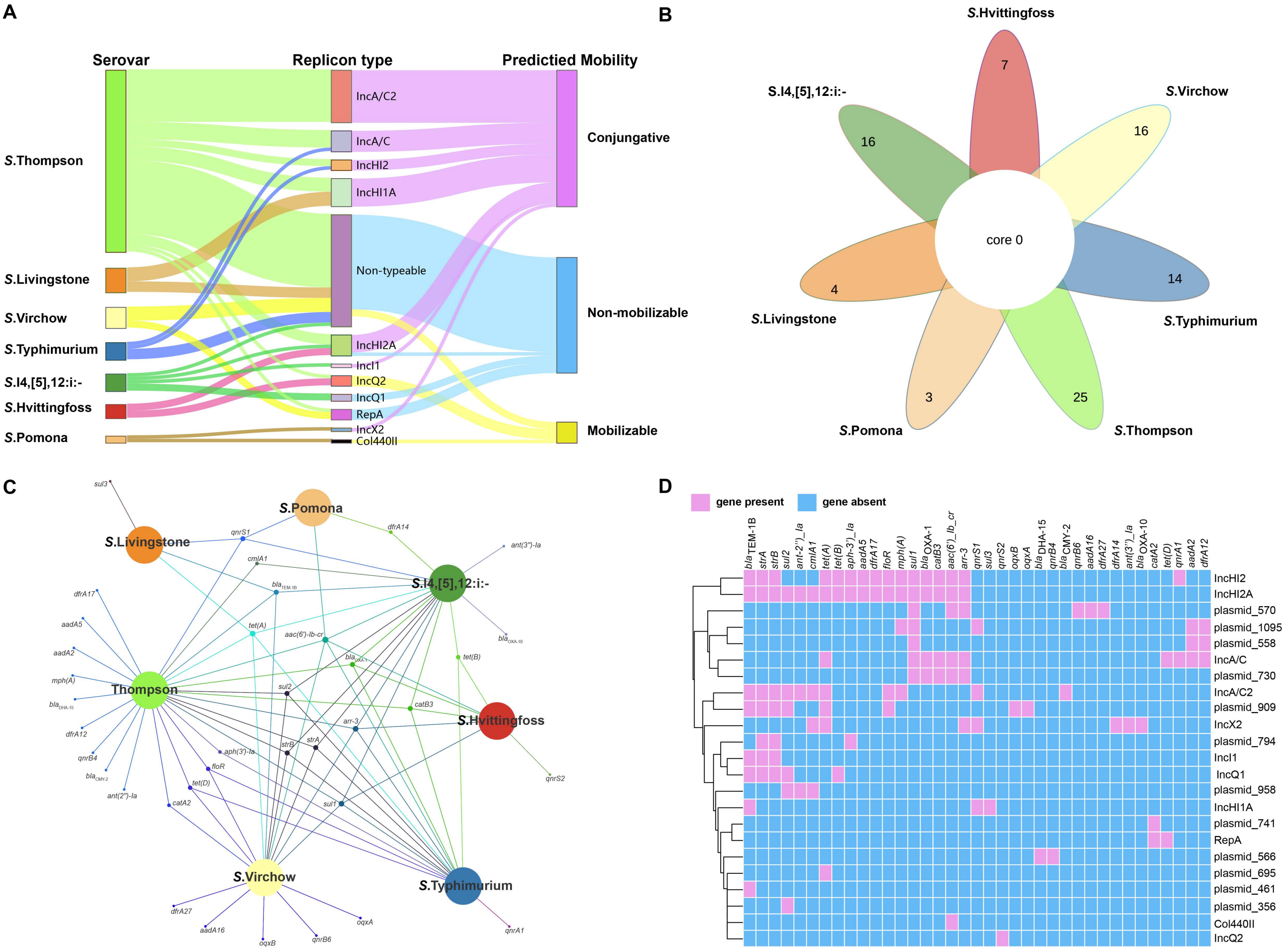

Figure S2. (A) Sankey diagram exhibiting the serovars, replicon types and predicted mobility of resistant plasmids detected from pet turtle-associated *Salmonella* isolates in this study. (B) Flower diagram exhibiting the numbers of AMR genes detected from pet turtle-associated *Salmonella* isolates of different serovars. (C) AMR genes shared across pet turtle-associated *Salmonella* isolates of different serovars. (D) Heatmap of presence (lavender pink) or absence (sky blue) of AMR genes identified from pet turtle-associated *Salmonella* isolatesco-occurred with different plasmid replicon types in this study.

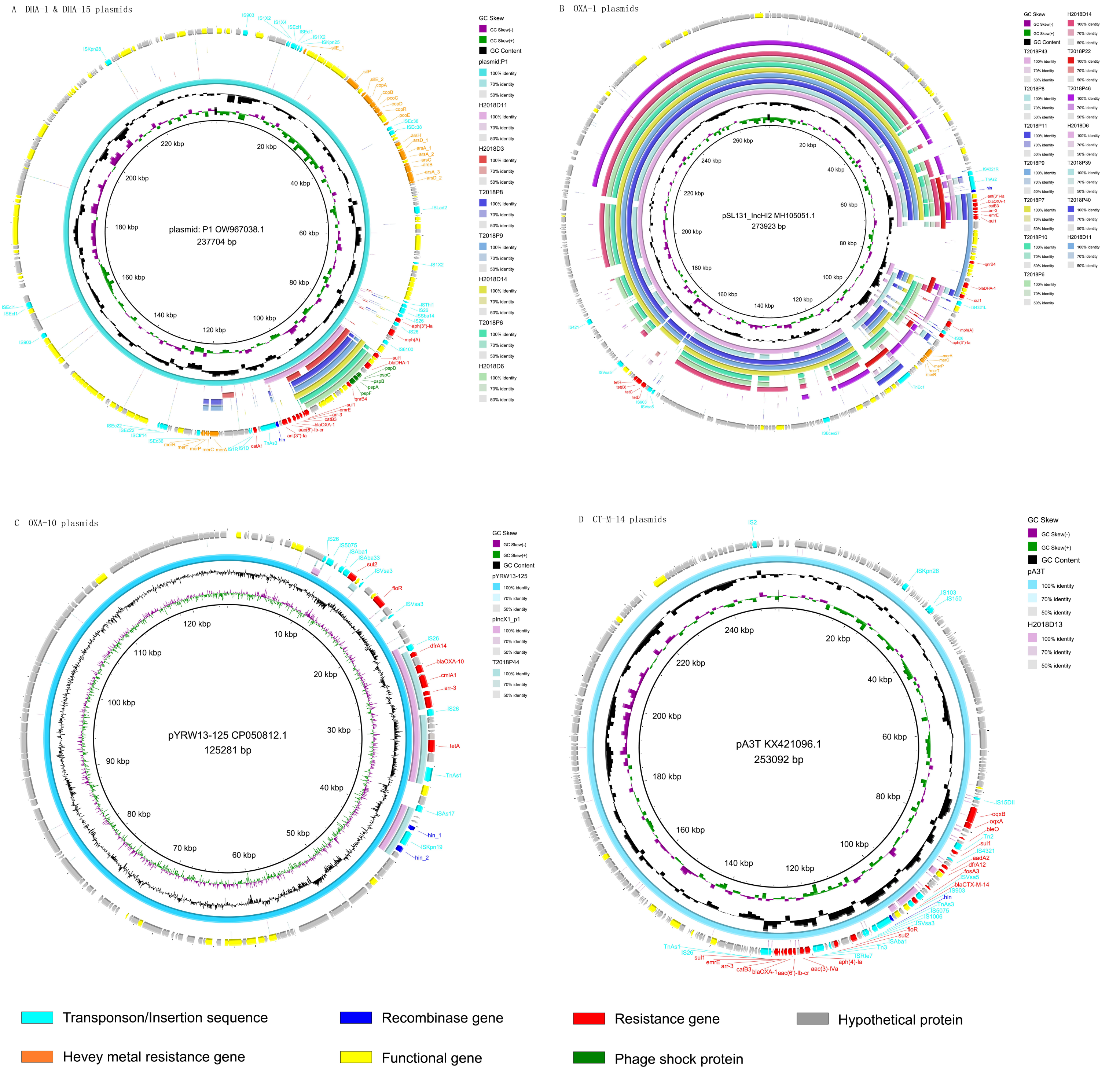

Figure S3. Comparison between the DHA-1 and DHA-15 (A), OXA-1 (B), OXA-10 (C) and CTX-M-14 (D) genes in this study and those in the online NCBI database. Contigs of potentially plasmid derived and reference plasmids are color-coded in the rings from the inside out. Transposons/Insertion sequences, recombinase genes, resistant genes, hypothetical genes, heavy metal resistant genes, functional genes and phage shock proteins are color-coded and labeled in the outmost ring indicating the reference plasmids used for comparison.
